# Psychometric validity and clinical utility of the GHQ-12 for screening psychological health in isolated, confined and unusual or extreme environments

**DOI:** 10.4102/ajopa.v8i0.195

**Published:** 2026-07-24

**Authors:** Charles H. van Wijk, Chris J.B. Muller

**Affiliations:** 1 Division of Health Systems and Public Health, Department of Global Health, Faculty of Medicine and Health Sciences, Stellenbosch University, Tygerberg, South Africa; 2 Department of Statistics and Actuarial Science, Faculty of Economic and Management Science, Stellenbosch University, Stellenbosch, South Africa

**Keywords:** grey-zone thresholds, ICUE environments, occupational mental health, resilience, screening, South Africa, validity

## Abstract

This study evaluated the psychometric validity and clinical utility of the 12-item General Health Questionnaire (GHQ-12) in isolated, confined and unusual or extreme (ICUE) work environments in South Africa. Two occupational health surveillance cohorts (*N* = 730 and *N* = 128) representing diverse occupational fields completed the GHQ-12 together with validated measures of common mental disorders, psychological resilience, emotional wellbeing and mental toughness. Confirmatory factor analyses supported a bifactor model comprising a strong general factor with minimal unique variance attributable to the positive and negative item subfactors. Internal consistency was high across samples. The GHQ-12 total scores correlated positively with depression, anxiety and adjustment disorder symptoms, and negatively with psychological resilience, emotional wellbeing and mental toughness, supporting convergent validity. Test–retest reliability was moderately strong, with a minority showing marked deterioration of mental health under more stressful operational conditions, indicating the tool’s potential sensitivity to environmental stressors. The GHQ-12 total scores differentiated between individuals with and without common mental disorders, and receiver operating characteristic analyses indicated acceptable sensitivity and specificity at optimal thresholds. Introducing a ‘grey-zone’ approach with lower and upper thresholds enhanced its practical application for surveillance and triage, by improving risk identification and decision-making for occupational health practitioners.

**Contribution:**

This study provides the first large-scale evaluation of the GHQ-12 in ICUE occupational environments in southern Africa. By combining psychometric evaluation with a clinically oriented grey-zone approach, it offers evidence to support how brief mental health tools can be applied to high-risk workplaces such as offshore vessels, submarines and polar research stations.

## Introduction

Effective brief and broad-based screening for psychological health in large samples is critical to support workplace mental health and wellbeing. The General Health Questionnaire (GHQ) is concise, widely used and inexpensive, offering promise as a useful screening tool within occupational health surveillance programmes, particularly in isolated, confined and unusual or extreme (ICUE) work environments.

### General Health Questionnaire

The original GHQ consisted of 60 items, and while many shorter versions exist, the 12 item GHQ appears most widely used. The popularity of the GHQ-12 can be attributed to its robust psychometric properties and being quick and unobtrusive to administer (Goldberg et al., 1997; Goldberg & Hillier, 1979; Goldberg & Williams, 1991; Wojujutari et al., 2024). The 12-item GHQ (Goldberg & Williams, 1991) aims to assess psychological distress by targeting the least-differentiated level of mental illness (that is, the lowest common denominator, shared by most psychiatric diagnoses). It can also be used to quantify psychological adaptability – on a mental wellbeing versus mental distress continuum – by measuring short-term changes in mental health and levels of psychological functioning across fixed time periods. The GHQ-12 comprises six positively and six negatively phrased items (Goldberg et al., 1997).

Good reliability and validity have been reported across international studies, including in worker samples (Hankins, 2008; Makowska & Merecz, 2000; Makowska et al., 2002; Wojujutari et al., 2024). Additionally, studies reported high validity in developing countries, with little age, gender, ethnic and educational level effect on validity observed across countries and cultures (Goldberg et al., 1997; Habibi Asgarabad et al., 2023; King et al., 2023; Liu et al., 2023; Wojujutari et al., 2024). Furthermore, there is substantial evidence of its *clinical utility* across contexts (cf. Wojujutari et al., 2024 for overview). South African population norms are not yet available.

The GHQ-12 was originally intended as a unidimensional measure, but evidence has been reported for 2-, 3- and 4-factor, as well as bifactor models (cf. Wojujutari et al., 2024, for summary). While the superiority of any model over another is still under debate (Hystad & Johnsen, 2020; Wojujutari et al., 2024), the current weight of evidence favours a bifactor model (Centofanti et al., 2019; Gnambs & Staufenbiel, 2018; Hystad & Johnsen, 2020). In this model, the GHQ-12 comprises a general factor that accounts for most of the observed variance, with two specific orthogonal latent factors (reflecting the six positively and six negatively phrased items) adding some additional variance attributed to wording effects.

The most common explanation proposes that the variance in the two latent factors reflect an artefact of the directionality of item wording (Centofanti et al., 2019; Gnambs & Staufenbiel, 2018; Hystad & Johnsen, 2020). Both positive and negatively phrased items are often included in questionnaires to reduce response bias, but risks introducing method variance, which refers to the creation of artificial factors based on response style. Such occurrence of pseudo-psychometric factors largely determined by item valence is not uncommon in multilingual samples, also encountered in South Africa (SA; Arendse et al., 2020; Van Wijk, 2024).

Current evidence suggests that the two subfactors do not represent independent constructs beyond the general factor, with the general factor explaining between 60% and 90% of combined variance across studies (Centofanti et al., 2019; Gnambs & Staufenbiel, 2018; Hystad & Johnsen, 2020). Subscale-specific variance associated with wording of items therefore appear negligible (Gnambs & Staufenbiel, 2018).

Hu and colleagues (Hu et al., 2007) offered evidence for an alternative interpretation of the two subfactors. In their understanding, the two factors represent two largely independent, though correlated, dimensions of mental health and psychological functioning, termed Symptoms of Mental Distress (SMD) and Positive Mental Health (PMH). This alternative model has the advantage of offering a more comprehensive description of respondents’ psychological health, by drawing on symptoms of psychopathology as well as indicators from positive psychology. It views the two factors as related but somewhat independent constructs, rather than as an artefact of language-related response patterns.

Despite robust psychometric support for the GHQ-12, a recent meta-analysis clearly illustrated the heterogeneity in factorial models and significant variability in reliability estimates across studies (Wojujutari et al., 2024). The authors of the meta-analysis recommended further exploration of *context-awareness* in the application of the questionnaire (Wojujutari et al., 2024). This recommendation formed the basis of the current study, namely, to consider evidence of validity in the specific context of ICUE environments in a sample consisting of naval and other maritime industries’ personnel.

### Isolated, confined and unusual or extreme work environments

Isolated, confined and unusual or extreme environments refer to settings characterised by physical isolation, spatial confinement and demanding or hazardous operational conditions that may require specialised technologies to sustain human functioning. Examples include underwater habitats and submarines, spacecraft, remote polar or meteorological stations and many forms of maritime deployments at sea (Suedfeld & Steel, 2000; Van Wijk & Martin, 2021). Personnel operating in such environments are exposed to distinctive and context-specific combinations of physical, psychological and social stressors that can place sustained demands on both individual and group functioning. Effective psychological adaptation is therefore essential for maintaining personal wellbeing, operational effectiveness and interpersonal stability in these contexts (Palinkas et al., 2011; Sandal et al., 2006).

Interest in psychological functioning within ICUE environments has increased in southern Africa because several strategically and economically important industries require personnel to operate under isolated and demanding conditions. For example, the SA Deep-Sea Trawling Industry (https://sadstia.co.za/), which operates numerous deep-sea vessels, is worth about 6 billion rand annually. Offshore oil and gas activities rely on drilling platforms and support vessels operating along the southern African coastline, extending from Angola to Mozambique The South African Navy further operates submarines and long-range patrol vessels (https://en.wikipedia.org/wiki/South_African_Navy). Although these settings vary in severity and operational purpose, all involve degrees of isolation, confinement, and while not all are necessarily extreme, they are undoubtably unusual and demanding.

The psychological demands associated with ICUE environments often extend beyond ordinary occupational stressors. Personnel may be required to maintain prolonged vigilance, perform complex tasks under high levels of responsibility and operate specialised life-support systems in contexts where errors can have serious consequences. Additional psychosocial challenges include restricted communication with the outside world, cramped living spaces, enforced intimacy with individuals not of one’s choosing, and navigating evolving group dynamics and emotional isolation (Sandal, 2000, p. A37; Van Wijk, 2023, pp. 1–2).

Isolated, confined and unusual or extreme environments place greater demands on individuals’ and groups’ adaptive functioning capacities compared to more conventional environments (Palinkas & Suedfeld, 2008; Sandal, 2000; Shea et al., 2009). Isolation and confinement also decrease an individual’s ability to regulate emotions (Liu et al., 2016), making people in ICUE settings vulnerable to health problems, reduced emotional wellbeing, decreased performance and interpersonal tension (Basner et al., 2014; Palinkas & Suedfeld, 2008; Sandal, 2000; Shea et al., 2009). Results from general population studies cannot be applied to ICUE workplace samples without due consideration of the potentially unique challenges to coping and wellbeing in unusual and demanding environments.

### Rationale and aims

The GHQ-12 was previously used in ICUE environments, where split-scores based on population median distinguished between optimal and sub-optimal psychological adaptation (cf. Le Roy et al., 2024 for summary). It has also been used in naval ships (cf. Hystad & Johnsen, 2020, for summary; Sanden et al., 2014). The bifactorial structure observed in the Hystad and Johnsen (2020) study corresponded to the negatively and positively phrased items, and they recommended that the scale be considered unidimensional for *practical* purposes in related ICUE contexts.

The current study reports on indicators of psychometric validity and clinical utility of the GHQ-12 to screen for psychological health in ICUE environments. Two broad aims guided the analysis: The first aim was to offer evidence of psychometric validity in a sample from an ICUE context. This aim was pursued through three specific objectives:

- To replicate previous studies that assessed structural validity, through confirmatory factor analysis (CFA) and internal consistency analysis, in ICUE environments.
- To extend previous investigations of the screening of psychological health by evaluating convergent validity, through correlating GHQ-12 scores with other psychological measures, in ICUE environments.
- To extend previous investigations of the protective effect of resilience and social support, and to consider the relative contribution of variables with significant associations to GHQ-12 scores.

The second aim was to explore the clinical utility of the GHQ-12 to identify poor psychological health for practical application, and to introduce grey-zone scores as a potential mechanism for risk identification in ICUE and other resource-restricted settings.

## Methods

### Participants

*Sample 1* consisted of 730 South Africans working in ICUE environments. The data come from medical records of full-time employees who participated in employer-sponsored occupational health surveillance initiatives over a specified 12-month period (during 2023–2024). The group represented diverse vocational fields, including technical engineering (23.5%), maritime warfare (e.g. sonar, radar, weapons, communications; 22.5%), clerical and administrative (15.5%), security (13.5%) and marine officers (5.5%). All participants were skilled workers on formal contracts. Cases were eligible if documented consent for use of their psychological data was available, if data for the GHQ-12 and socio-demographic variables (age, gender and language) were complete, and if participants completed high school and formal vocational training. Further, all participants had to self-report as proficient in English. All eligible cases were included in the dataset, and all data were de-identified during extraction from the archives.

The sample had a mean age of 35.3 (± 9.5, range: 20–60), of which 33.2% were women and 66.8% were men. English as home language was spoken by 19.0% of the sample, while the rest reported one of the other 10 SA official spoken languages as mother tongue.

In addition to the GHQ-12, participants in Sample 1 also completed the measures of common mental disorders (CMD) as well as of mental health and wellbeing described below. The outcome from an interview with a psychologist (in the form of diagnostic codes for CMD) were also available.

*Sample 2* consisted of 128 South Africans engaged in long-range fishery law-enforcement patrols, with a mean age of 32.0 (± 6.2, range 21–51), and comprising 21% women and 79% men. Participants completed the GHQ-12 on the day of their departure (together with MTQ-6 and Brunel Mood Scale [BRUMS]), and again on the day of their return from a 3-week patrol (*n* = 105 at retest). Additionally, a subsample of 48 completed the GHQ-12 again after a second 3-week patrol (51 days after the first administration). Cases were included in the study if complete data for the three scales, as well as documented consent for its use, were available.

The two samples served partially different functions within the study design. Sample 1 was used primarily to evaluate structural validity, convergent validity with CMD measures, risk/protective factors and clinical utility analyses. Sample 2 was used primarily to examine temporal stability and change sensitivity across repeated operational deployments, as well as convergent validity with measures of emotional wellbeing and mental toughness. Analyses were conducted separately for each sample, and no pooled inferential analyses were performed across the two cohorts.

### Measures

All measures were administered in their standard English format.

#### General Health Questionnaire

The GHQ-12 includes six positively phrased items and six negatively phrased items, each with four answer options (Goldberg et al., 1997). Two scoring systems are in common use:

- The Traditional (also referred to as Clinical) binary scoring method (0 – 0 – 1 – 1) indicates presence/absence of symptoms. Scores are summed, and a threshold of ≥ 3 is typically used to indicate significant mental distress (Armino et al., 2021).
- The Likert scale method (0 – 1 – 2 – 3) indicates severity of reported symptoms. Scores are again summed, with higher scores indicative of lower psychological wellbeing. A range of thresholds can be used to indicate significant mental distress. For example, older studies proposed a score of 11 or 12 as neurotypical, with scores > 15 associated with psychological distress; and scores > 20 with severe psychological distress in general population samples (Goldberg & Williams, 1991; Shevlin & Adamson, 2005). In more recent studies, the optimal threshold for general mental health problems appear to cluster around 10–12 (cf. Table 1 in Anjara et al., 2020, for summary). The Likert scale method was used in the current study, to examine finer differentiation across multiple administrations.

**TABLE 1:** Descriptive statistics of measures across two samples.

Measure	*n*	Mean	s.d.	Median	Range	McDonald’s *ω*
**General ICUE sample (Sample 1)**						
GHQ-12	730	5.62	4.25	6.00	0–27	0.894
PHQ-9	730	1.85	3.18	0.00	0–24	0.861
GAD-7	730	1.31	2.67	0.00	0–19	0.896
IADQ	729	1.32	3.52	0.00	0–24	0.943
CD-RISC-10	725	32.27	5.61	32.00	10–40	0.907
BRUMS	406	−6.07	9.02	−8.00	−16 to 53	-
**Seaward sample (Sample 2)**						
GHQ-12	128	6.02	4.14	6.00	0–24	0.818
MTQ-6	128	25.43	2.65	26.00	17–30	0.806
BRUMS	128	−4.48	8.26	−6.00	−16 to 23	-

BRUMS, Brunel Mood Scale; GAD, General Anxiety Disorder scale; GHQ, General Health Questionnaire; CD-RISC-10, Connor–Davidson Resilience Scale; IADQ, International Adjustment Disorder Questionnaire; MTQ, Mental Toughness Questionnaire; PHQ, Patient Health Questionnaire; s.d., standard deviation; ICUE, isolated, confined and unusual or extreme.

#### Validated measures of common mental disorders

Sample 1 completed three measures of CMDs. The *Patient Health Questionnaire-9 (PHQ-9;* Gilbody et al., 2007) was used to screen for depressive symptoms. The *Generalised Anxiety Disorder-7 (GAD-7;* Löwe et al., 2008) was used to screen for symptoms of generalised anxiety. The *International Adjustment Disorder Questionnaire (IADQ)* was developed by the WHO to enable cross-national application of adjustment disorder (AjD) screening (Shevlin et al., 2020). It consists of five sections, reflecting the updated ICD-11 diagnostic criteria for AjD. A symptom severity score can also be calculated (Shevlin et al., 2020) and was used in this study.

#### Validated measures of mental health and wellbeing

Sample 1 also completed two additional measures of mental health and wellbeing. The *Connor–Davidson Resilience Scale-10* (*CD-RISC-10*; Campbell-Sills & Stein, 2007; Pretorius & Padmanabhanunni, 2022) was used to measure psychological resilience. The *Brunel Mood Scale* is a measure of emotional wellbeing that taps transient affective mood states (Terry et al., 2003). The 20-item BRUMS (without the Confusion subscale) was completed by a subsample (*n* = 406). The total mood distress score (Terry et al., 2003) – where higher scores represent poorer emotional regulation – was used here. Sample 2 also completed the BRUMS, as well as the *Mental Toughness Questionnaire-6 (MTQ-6*; Kawabata et al., 2021), a brief measure of mental toughness, which is a construct associated with psychological resilience and perseverance in the face of adversity.

#### Other markers

**Mental health history questionnaire:** Sample 1 completed a mental health history questionnaire that included: a history of previous CMD diagnosis and treatment, a history of personal traumatic experience (using the DSM-5 definition), as well as recent social support (assessed by self-reported close emotional connection with partner/immediate family and availability of support from close family/friends).

**Clinical interview (Sample 1 only):** Clinical interviews were conducted by registered psychologists working within the occupational health programme. Interviews took place later on the same day as the administration of the occupational mental health screening battery. Diagnostic coding followed standardised organisational procedures aligned with DSM-5 and ICD-11 criteria criteria and were available in the medical records. The practitioners all had extensive experience in this field and also met monthly to support procedural and diagnostic consistency. Interviews formed part of routine clinical care, and no independent diagnostic verification was conducted for this study. Practitioners were blind to GHQ-12 scores.

### Data analysis

R was used to conduct structural analyses. IBM^®^ SPSS^®^ for Windows, Version 29, were used for all other analyses. Prior to conducting inferential analyses of Sample 1 data, relevant statistical assumptions were examined and found to be adequately met. Visual inspection of histograms and Q-Q plots indicated some skewness (+1.299; tail to right), with Shapiro–Wilk test significant (*p* > 0.05). However, the deviation was considered acceptable, and analyses proceeded due to robustness of parametric tests with large samples.

#### Descriptive statistics

The effect of age was examined with Pearson’s correlation, and gender and language with *t*-tests for independent samples. Gender was coded into two groups (*women, men*) based on participant self-identification. Participants’ self-reported home language was also coded into two groups (*English as home language, not-English as home language*). Sample 1 data were used to examine age, gender and language effects. Although descriptive statistics for both cohorts are presented together in Table 1 for comparative purposes, inferential analyses were conducted separately for each sample.

#### Structural validity

Structural analyses, using Sample 1 data, were conducted in R version 4.4.2 (R Core Team, 2023). Dimensionality was assessed with CFA, which is used to evaluate whether the data fit a hypothesised measurement model. Item responses were treated as ordinal variables. The package lavaan (v06-1) was used to fit the CFA models, and all models were estimated using Diagonally Weighted Least Squares. McDonald’s ω (categorical ω) was calculated using the package MBESS (v4.9.3). Following previous studies, four models were tested:

- A unidimensional model where all 12 items load onto one construct.
- A model with two correlated latent factors, as originally described by Andrich and Van Schoubroeck (1989), with the two latent constructs also representing the positive and negatively phrased items (6 items respectively).
- A three-factor model originally described by Graetz (1991), consisting of Anxiety/Depression (4 negatively phrased items), Social Dysfunction (6 positively phrased items) and Loss of confidence (2 negatively phrased items).
- A bifactor model, as recommended by Gnambs and Staufenbiel (2018), consisting of a general factor and two specific orthogonal latent factors (representing the positive and negatively phrased items, respectively).

For a CFA, the global fit χ^2^ would ideally be small and not significant, but this is rarely achieved in samples above 400, and the following indices with cut-off points were also taken into consideration: a root mean square error of approximation (RMSEA) between 0.05 and 0.08 suggests a reasonable approximate fit, the comparative fit index (CFI) should be > 0.90, the Tucker-Lewis index (TLI) > 0.95, and the standardised root mean square residual (SRMR) should be < 0.08 (Hu & Bentler, 1999; Kline, 2016). To overcome the potential drawback of Cronbach’s α, internal consistency was examined with McDonald’s ω, where results ≥ 0.80 indicating good reliability (Dunn et al., 2014; Hayes & Coutts, 2020; Kelley & Pornprasertmanit, 2016)

Previous studies tested for measurement invariance in GHQ responses, for gender (Habibi Asgarabad et al., 2023; Liu et al., 2023) and ethnicity (King et al., 2023), and measurement invariance testing was planned for gender and language in Sample 1. However, due to insufficient representation of some groups across the variables that resulted in empty cells in the data matrix, the estimation of measurement invariance could not be performed.

#### Convergent validity

Convergent validity was evaluated using bivariate correlation analyses of total and subscale scores with other measures of CMD (namely PHQ-9, GAD-7, IADQ) and mental health and wellbeing (namely CD-RISC-10, BRUMS, MTQ-6). A correlation above 0.30 was considered as moderate, and above 0.50 as strong. Data of Samples 1 and 2 were analysed separately.

#### Temporal stability and operational change sensitivity

Test–retest reliability, using Sample 2 data, was evaluated with a paired sample *t*-test, using data collected 21 and 51 days after first administration. In the administration to Sample 2, no substantial variations in GHQ-12 total scores across the first 21 days were expected, with scores anticipated to remain relatively stable throughout the mission duration (cf. Bell & Garthwaite, 1987). As all personnel had similar environmental exposure during the uneventful 3 weeks, relatively adaptive psychological adjustment was expected. In contrast, the second 21-day period was not uneventful, with severe weather conditions and mechanical problems providing different participants with different experiences (in terms of workload, motion sickness, etc.), and GHQ-12 changes across administrations would not have been unexpected.

#### Risk and protective factors

Previous investigations into the role of resilience and/or social support were extended in this study by considering the relative contribution of all variables with significant associations to GHQ-12 scores, using Sample 1 data. These variables included age, gender, resilience scores, history of previous mental health concerns, history of previous trauma exposure and social support (both with immediate family, and availability of support).

The effects of socio-demographic and psychological variables were first investigated through *t*-test for independent samples (for gender, history of mental disorder, history of psychotherapy, history of personal traumatic exposure and the two social support indicators) and bivariate correlations (for age and resilience scores). Significant variables were then entered into a multiple regression analysis (MRA) to determine their relative contributions to GHQ-12 scores.

#### Clinical utility

Clinical utility was examined through a number of analyses drawing on Sample 1 data. First of all, the GHQ-12’s ability to differentiate between individuals with major depressive disorder (MDD), GAD, AjD and those without was assessed using *t*-test for independent samples. A Receiver Operator/Operating Characteristic (ROC) curve analysis was also conducted and were reported as Area under the Curve (AuC), to determine the GHQ-12’s ability to predict the presence of CMD (i.e. MDD, GAD, AjD). Youden’s Index was used to determine the respective optimal cut-off point’s sensitivity and specificity.

However, the use of rigid cut-off scores with scales of psychological health can be problematic, given the potential influence of intra-individual and situation-specific conditions on questionnaire responses. A ‘grey-zone’ approach (Cannesson et al., 2011; Coste & Pouchot, 2003) was used to address this. The grey zone refers to the space between a lower threshold score that maximises sensitivity (the ‘at-risk’ threshold – interpreted as requiring closer monitoring) and an upper threshold score that maximises specificity (the ‘intervention’ threshold – interpreted as requiring action). The grey-zone approach functions as a triage heuristic that supports graded decision-making rather than binary classification and was applied to GHQ-12 total scores to enhance its practical application for decision-making in clinical and occupational health settings. As thresholds are context-dependant, illustrative cut-offs for Sample 1 were defined as a sensitivity of 95% for the lower threshold and a specificity of 95% for the upper threshold. This approach aligns with dimensional models of psychopathology and could support proportionate decision-making in occupational settings.

Analyses proceeded in five stages. Firstly, descriptive and structural analyses were conducted using Sample 1 data to evaluate dimensionality and internal consistency. Secondly, convergent validity analyses were performed using psychological measures available within each sample. Thirdly, repeated-administration analyses in Sample 2 evaluated temporal stability and sensitivity to operational stress exposure. Fourthly, Sample 1 data were used to examine psychosocial risk and protective factors associated with GHQ-12 scores. Finally, clinical utility analyses, including ROC and grey-zone analyses, were conducted using Sample 1 diagnostic outcomes.

### Ethical considerations

Participants consented that their data, in anonymised form, could be used for this study, and this was recorded in their medical records. This specific retrospective record review and analysis of anonymised clinical data was approved by the Health Research Ethics Committee of Stellenbosch University (#N25/05/046). All data were deidentified prior to inclusion in the study dataset.

## Results

### General description and socio-demographic effects

**FIGURE 1:**
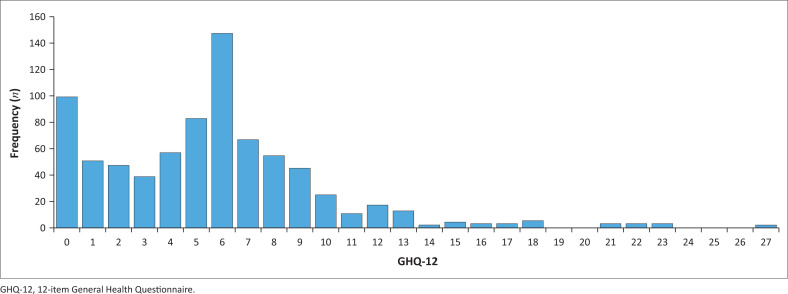
Distribution of 12-item General Health Questionnaire scores for Sample 1.

The GHQ-12 total score distribution of both samples is presented in Table 1 and graphically represented in Figure 1. Bartlett’s test of sphericity (3216.680, *p* < 0.001), supplemented by the Kaiser–Meyer–Olkin measure of sample adequacy (0.885), was applied to Sample 1 and indicated that the data satisfied the assumptions for factor analysis.

In Sample 1 (*N* = 730), there was no significant correlation between age and GHQ-12 total scores (*r* = –0.013, *p* = 0.725). There was a significant but small mean difference between GHQ-12 total scores for women and men (*t* = –3.063, *p* < 0.01, Cohen’s *d* = 0.24), with women scoring 1 point higher. There was also a significant but small mean difference between GHQ-12 total scores of English first language and non-English first language speakers (*t* = 2.327, *p* < 0.05, Cohen’s *d* = 0.24), with English first language speakers scoring 1 point higher.

### Structural validity

#### Dimensionality

In order to optimise model convergence and enhance statistical robustness in a sample potentially not normally distributed (see Figure 1), standard scores were reported for the CFA. The detail statistics can be found in Table 2. The unidimensional model was a poor fit to the data. There was some support for the 3-factor model, while the 2-factor model showed acceptable fit (with individual factors strongly correlated to each other; *r* = 0.576). The bifactor model appeared the best fit to the data. The two subfactor scores were strongly correlated to each other (*r* = 0.593), and they were also individually correlated with the total scale scores (SMD: *r* = –0.282, PMH: *r* = –0.571).

**TABLE 2:** Goodness of fit statistics.

Model	*χ* ^2^	*df*	*χ*^2^/*df*	*p*	CFI	TLI	RMSEA	90% CI	SRMR
Unidimensional	1170.550	54	21.68	<0.001	0.823	0.805	0.168	0.160–0.177	0.141
2-factor	151.965	53	2.87	<0.001	0.939	0.936	0.061	0.051–0.070	0.052
Bifactorial	27.546	39	0.71	0.915	1.000	1.000	0.000	0.000–0.010	0.023
3-factor	150.270	51	2.95	<0.001	0.909	0.905	0.062	0.052–0.071	0.055

CFI, comparative fit index; TLI, Tucker-Lewis index; RMSEA, root mean square error of approximation; CI, confidence interval; SRMR, standardised root mean square residual; *df*, degree of freedom.

#### Internal consistency

The GHQ-12 showed acceptable internal reliability in both samples. McDonald’s ω for the total scale in Sample 1 was 0.894 (SMD = 0.736; PMH = 0.958), and 0.818 in Sample 2. No item deletions improved the coefficients in either sample.

### Convergent validity

The GHQ-12 correlated positively and significantly with measures of CMD, and negatively and significantly with measures of resilience (but with small effect size) and emotional wellbeing, across both samples. The detail is presented in Table 3.

**TABLE 3:** Correlation between 12-item General Health Questionnaire and other measures of distress and wellbeing.

Measures	PHQ-9 (*N* = 730)	GAD-7 (*N* = 730)	IADQ (*N* = 729)	CD-R-10 (*N* = 724)	BRUMS (*N* = 406)	MTQ-6 (*N* = 128)
**GHQ-12 (*N* = 730)**	0.559**	0.571**	0.559**	−0.355**	0.694**	-
SMD	0.665**	0.689**	0.653**	−0.358**	0.716**	-
PMH	0.286**	0.283**	0.299**	−0.243**	0.469**	-
**Sample 2 (*N* = 128)**	-	-	-	-	0.654**	−0.383**
SMD	-	-	-	-	0.616**	−0.441**
PMH	-	-	-	-	0.504**	−0.216*

BRUMS, Brunel Mood Scale; GAD, General Anxiety Disorder scale; GHQ, General Health Questionnaire; IADQ, International Adjustment Disorder Questionnaire; MTQ, Mental Toughness Questionnaire; PHQ, Patient Health Questionnaire; PMH, positive mental health; SMD, symptoms of mental distress.

* , *p* < 0.05;

** , *p* < 0.001.

### Temporal stability and operational change sensitivity

A subsample of 105 sailors from of Sample 2 completed the GHQ-12 again after 21 days, with a strong correlation (*r* = 0.598, *p* < 0.001) and no significant mean score difference (range 0–8; *t* = –0.759, *p* = 0.450; mean difference [diff] = 0.27).

Additionally, a subsample of 48 sailors completed the GHQ-12 again after the second 21-day demanding deployment, with a total time between first and last administrations of 51 days. The test–retest coefficient was moderate (*r* = 0.336, *p* = 0.020) and again with no significant mean score differences (range 0–20; *t* = –1.143, *p* = 0.259; mean difference = 0.77). While this still suggests a reasonable retest reliability, closer examination of the range of retest score differences across the two time points offers a more nuanced description, as detailed in Table 4. The low mean score difference provides evidence of longer-term temporal stability for most participants. However, the larger score changes (≥ 8 points) observed in a smaller minority suggest that the GHQ-12 may simultaneously demonstrate discriminative utility, in that it could identify individuals who reported deterioration in mental health following their deployment experience.

**TABLE 4:** Total 12-item General Health Questionnaire score differences after 21 and 51 days.

Score difference	21 days (%) (*n* = 105)	51 days (%) (*n* = 48)
No change in total scores or improved psychological health	77.2	72.9
Decreased psychological health (≥ 7 points from baseline)	21.8	18.8
Substantially decreased psychological health (≥ 8 points from baseline)	1.0	8.3

### Risk and protective factors

The significant effect of gender and psychological resilience has already been reported for Sample 1. Moreover, in Sample 1, GHQ-12 total scores differentiated between individuals with histories of mental disorder (*t* = –4.720, *M* difference = 5), psychotherapy (*t* = –9.801, *M* diff = 4.5) or previous personal traumatic exposure (*t* = –5.534, *M* diff = 2.5), as well as a close emotional connection with immediate family (*t* = –9.535, *M* diff = 4.5), and individuals without (< 0.001 for all). These variables were therefore entered into an MRA, which significantly predicted GHQ-12 scores (*F*_7,715_ = 36.358, *p* < 0.001, *R*^2^_ajd_ = 0.255). The overall model explained a modest 25% of the variance. The statistical results are presented in Table 5.

**TABLE 5:** Multiple regression analysis outcome.

Variable	*β*	*t*	*p*-value
Gender	0.033	1.011	0.312
History of mental disorder	0.031	0.914	0.361
History of psychotherapy	0.219	6.232	< 0.001
History of traumatic exposure	0.078	2.332	0.020
Connection with immediate family	0.244	7.399	< 0.001
Availability of emotional support	−0.025	−0.773	0.440
Psychological resilience	−0.255	−7.662	< 0.001
(Constant)	-	11.365	< 0.001

A history of personal traumatic exposure did pose a statistically significant a risk but practically may have less effect. A history of previous psychotherapy, close connection with immediate family and higher scores on measures of psychological resilience appear to offer meaningful protection against mental distress as measured by the GHQ-12.

### Clinical utility

In Sample 1, the GHQ-12 total scores differentiated between individuals with MDD (16 cases, 2.2% of sample) and those without (*t* = –10.401, *p* < 0.001, Cohen’s *d* = 2.63, *M* diff = 10.4). It also differentiated between individuals with GAD (11 cases, 1.5% of sample) and those without (*t* = –8.239, *p* < 0.001, Cohen’s *d* = 2.50, *M* diff = 10.2). Lastly, it differentiated between individuals with AjD (22 cases, 3.0% of sample) and those without (*t* = –11.683, *p* < 0.001, Cohen’s *d* = 2.53, *M* diff = 9.8).

Receiver Operator/Operating Characteristic curve analyses also indicated the usefulness of the GHQ-12 to identify cases of clinical concern. The detail statistics are presented in Table 6. The optimal cut-off was > 9 and > 10 for MDD and GAD, respectively. The grey-zone approach identified scores of > 7–8 as the lower threshold (‘require closer monitoring’) and scores of > 11–12 as the upper threshold (‘require urgent clinical attention’) for CMD. In all three cases, the AuC was acceptable but not excellent, and the sensitivity and specificity acceptable for research purposes. Against this finding, using grey-zone thresholds may be a more appropriate way to interpret GHQ-12 total scores. No different thresholds were found when analysing separate gender or language groups.

**TABLE 6:** Receiver Operator/Operating Characteristic curve analysis statistics.

Diagnosis	AuC	Optimal cut-off point	Sensitivity (%)	Specificity (%)	Lower threshold	Upper threshold
MDD	0.884	> 9	81.3	83.5	> 7	> 11
GAD	0.899	> 10	90.9	89.0	> 8	> 12
AjD	0.873	> 9	81.8	84.0	> 6	> 12

AuC, area under the curve; AjD, adjustment disorder; GAD, generalised anxiety disorder; MDD, major depressive disorder.

## Discussion

This study evaluated the psychometric validity and clinical utility of the GHQ-12 to monitor psychological health in ICUE environments. Using two occupational samples, the findings replicate and extend previous research on the factorial structure, convergent validity and discriminative ability of the GHQ-12 as a brief screen of psychological health in demanding occupational settings, as well as other resource-restricted mental healthcare contexts.

### General findings

The mean score of ± 6 was lower than the closest comparable ICUE sample (Le Roy et al., 2024) which reported a mean of 8, and substantially lower than the mean of 14 for seafarers on container ships during coronavirus disease 2019 (COVID-19) (Pesel et al., 2020). The mean score was also lower than general European population samples (e.g. *M* = 8.5; Sánchez-López & Dresch, 2008; *M* = 10.4; Giorgi et al., 2014) or Ghanaian nurses (*M* = 9; Opare-Asamoah et al., 2023). While the COVID-19 data reflected situational distress, the relatively lower mean scores found in the current study may partly reflect a sample that by virtue of their employment have demonstrated some adjustment in ICUE settings and partly reflect the impact of employer-supported occupational mental health care initiatives, from which the data were drawn. The 1-point higher mean score of women supports previous findings (Comotti et al., 2024). The variation in reported mean scores highlight the need for more narrowly defined context-specific normative data and cut-points, including in ICUE settings.

### Psychometric validity

The first aim of the study was to offer evidence of psychometric validity of the GHQ-12 in the context of ICUE work environments, to foreground later discussion on its clinical utility.

Confirmatory factor analyses supported a bifactor model consisting of a strong general factor accounting for most of the variance, and two orthogonal latent factors with negligible subfactor-specific variance, reflecting positive and negative item phrasing. This finding replicates existing meta-analytic evidence (Centofanti et al., 2019; Gnambs & Staufenbiel, 2018; Wojujutari et al., 2024) and further extends it to a multilingual African occupational sample working in ICUE contexts. This supports earlier recommendations to, for practical purposes, treat the GHQ-12 as essentially unidimensional when screening for psychological distress in ICUE environments (Hystad & Johnsen, 2020). Internal consistency was acceptable, and consistent with general reports (Wojujutari et al., 2024).

Although the subfactors showed different patterns of correlation with measures of mental distress and PMH, their limited unique variance suggests they add little additional information within routine surveillance. Nevertheless, they may hold conceptual value for describing the breadth of psychological functioning (cf. Hu et al., 2007) and warrant further study in ICUE contexts. While the two subfactors may be useful in therapeutic settings to describe emotions and behaviours associated with either PMH or mental distress, the unidimensional model may be more suitable for practitioners in settings where diagnostic cut-offs are used (Centofanti et al., 2019).

Convergent validity was demonstrated through strong positive correlations with measures of common mental disorders and moderate negative correlations with measures of resilience, emotional wellbeing and mental toughness.

### Clinical utility

With acceptable psychometric validity demonstrated in this sample, the second aim of the study was to explore the practical clinical utility of the GHQ-12 as a screening and monitoring tool in ICUE environments, and to introduce grey-zone interpretation as a structured mechanism for risk identification in resource-constrained settings.

The findings demonstrate that the GHQ-12 meaningfully differentiated between individuals with and without clinically identified psychiatric concerns. In applied settings, this capacity supports the GHQ-12’s use as a first-line triage instrument, enabling occupational health practitioners or other trained personnel to identify individuals who may require further psychological evaluation or intervention.

Beyond single-time-point screening, the longitudinal findings provide evidence for the GHQ-12’s role in ongoing monitoring of psychological health during deployments. Stability of mean scores across relatively uneventful operational periods suggests that the instrument does not generate excessive variability under stable conditions. However, the observation that a minority of individuals exhibited substantial score increases during more demanding operational periods highlights the GHQ-12’s sensitivity to environmental stressors. In practical terms, serial administration during extended periods in ICUE environments could function as an early warning mechanism, allowing individuals who show marked score increases to be flagged for closer monitoring or support before more severe psychological impairment emergences. The value of monitoring score changes during long-duration ICUE environment deployments have previously been demonstrated by Bell and Braitwaite (1987), who found that large increases in scores clearly identified cases of clinical concern in a group of Antarctic personnel.

The ROC analyses indicated optimal thresholds near 9–10 to indicate psychological distress. This is lower than the 11–12 suggested by some (Anjara et al., 2020; Goldberg et al., 1997), but higher than the 8–9 suggested by others (Le Roy et al., 2024; Politi et al., 1994), emphasising the need for context-specific normative data and cut-points. Reliance on a single rigid cut-off score may be additionally problematic in ICUE contexts, where environmental stressors fluctuate and access to mental health resources may be constrained. In response to this limitation, the grey-zone model offers a clinically meaningful framework for graded decision-making under conditions of uncertainty. Rather than classifying individuals as either distressed or not, the grey-zone model defines three operational categories: (1) scores below the lower threshold indicating routine functioning; (2) scores within the grey zone suggesting increased risk and the need for closer monitoring; and (3) scores above the upper threshold indicating a need for timely clinical intervention. This framework supports a tiered response approach: Individuals in the lower range may continue routine duties with periodic reassessment. Those in the grey zone may be scheduled for follow-up screening or informal support, while those exceeding the upper threshold require formal clinical assessment or referral to specialised care. Such graded interpretation aligns with contemporary stepped-care models of intervention and risk stratification and is particularly relevant in ICUE settings where immediate specialist support may be limited.

Beyond screening for distress, the utility of the GHQ-12 in ICUE environments lies also in its feasibility and scalability. Its brevity allows administration within constrained settings such as ships, offshore platforms and remote stations, where time, privacy and professional resources may be limited. Repeated administration across defined mission phases – for example pre-deployment, mid-deployment and post-deployment – may further enhance the detection of emerging distress trajectories that might otherwise remain undetected until more serious functional impairment occurs. Importantly, the grey-zone approach recognises the inherent uncertainty associated with psychological screening tools. In high-risk occupational environments, failing to identify distressed individuals may have more severe consequences than over-identification. A graded threshold system supports balanced decision-making, reducing both false reassurance and unnecessary referral. This approach is consistent with dimensional models of psychological health, which views distress as existing along a continuum rather than as a binary state.

Although further prospective validation is required, the absence of differential thresholds across gender and language groups suggests that a unified scoring framework may be feasible in multicultural occupational populations. This is consistent with findings of measurement invariance across demographic variables in other studies (Habibi Asgarabad et al., 2023; King et al., 2023) and may simplify implementation of the GHQ-12 across linguistically diverse workforces.

Taken together, the findings suggest that the GHQ-12 functions not simply as a screening instrument, but as a dynamic risk-monitoring tool capable of supporting staged decision-making in ICUE environments. When integrated into structured occupational health protocols, particularly in conjunction with grey-zone interpretation and serial monitoring, the GHQ-12 may contribute to earlier identification of psychological risk and more proportionate responses to emerging distress in settings where prevention, timely detection and operational safety are tightly interconnected.

Finally, the association of lower GHQ-12 scores with greater resilience and close family connections supports previous reports of the protective role of psychosocial resources in ICUE settings (Nordmo et al., 2020; Palinkas et al., 2011; Sandal, 2000) and offers potential avenues for mission preparation, such as resilience training or family liaison programmes.

### Limitations and future directions

This study has several limitations. Firstly, it relied mainly on cross-sectional self-report data, with only a subset of participants followed longitudinally, and no causality can be inferred. Further, given the small sample of psychologically distressed individuals, the ROC analysis outcome should be seen as preliminary and needs to be interpreted with caution. Secondly, measurement invariance by gender and language could not be formally tested due to small subgroup sizes, which limits the conclusions about applying uniform cut-offs across demographics. Thirdly, no South African population norms currently exist for the GHQ-12, limiting direct comparison with non-ICUE samples. Fourthly, clinical interviews were conducted as part of routine care and did not include independent diagnostic verification or inter-rater reliability assessment. Lastly, while the use of two occupational cohorts enabled examination of complementary aspects of validity and clinical utility, not all measures were available across both samples.

Future research should address these limitations by establishing local norms, conducting full invariance testing across demographic identifiers, and evaluating the GHQ-12’s predictive validity for long-term psychological health outcomes.

## Conclusion

The GHQ-12 demonstrated acceptable psychometric properties and promising clinical utility as a screening and monitoring tool for psychological health in ICUE environments. Its brevity, ease of administration and potential sensitivity to change under stressful conditions makes it attractive for large-scale occupational health surveillance. Integrating the GHQ-12 with a grey-zone interpretative approach into digital or real-time monitoring platforms could assist occupational health practitioners in the timely identification and support of at-risk individuals across diverse ICUE workplaces.
